# An experimental study: effects of boulder placement on hydraulic metrics of instream habitat complexity

**DOI:** 10.1038/s41598-022-17281-1

**Published:** 2022-08-01

**Authors:** Amir Golpira, Abul B. M. Baki, Haitham Ghamry, Christos Katopodis, Jonah Withers, David Minkoff

**Affiliations:** 1 Department of Civil and Environmental Engineering, Clarkson University, Potsdam, NY 13699 USA; 2 Freshwater Institute, Fisheries and Oceans Canada, 501 University Cres, Winnipeg, MB Canada; 3 Katopodis Ecohydraulics Ltd., 122 Valence Avenue, Winnipeg, MB Canada; 4 Lake Champlain Fish and Wildlife Resources Office, 11 Lincoln Street, Essex Junction, VT 05452 USA

**Keywords:** Restoration ecology, Civil engineering

## Abstract

Boulder placement is a common method to restore degraded instream habitats by enhancing habitat complexity. This experimental study is the foremost attempt to systematically investigate the influence of rock-ramp boulder placement with varying boulder concentration and flow rate on habitat hydraulic complexity metrics, including the kinetic energy gradient and modified recirculation metrics. By adding boulders to a reach, the modified recirculation metric increased by one order of magnitude for all boulder concentrations. Based on the studied metrics, boulder placement with the highest boulder concentration (λ = 8.3%) resulted in the greatest habitat hydraulic complexity. A set of relationships of moderate strength were proposed to predict the metrics in reaches with boulders by having information about only boulder concentration, habitat characteristic size, and reach-averaged flow characteristics. Based on the available data from the literature, boulder placement especially at higher concentrations may provide suitable habitats for several riverine fish species. Further studies are needed to establish a reliable linkage between the metrics and instream species, to test a wider variety of parameters for verifying and improving the range of applicability of the proposed relationships, and to find the structural configuration at which the habitat complexity is maximized or optimized for a certain species.

## Introduction

Numerous instream habitats throughout the world have been degraded due to factors such as sediment load changes, stream fragmentation (connectivity loss), and stream channelization (simplification)^1–3^. The resulting alteration of stream processes may negatively affect instream habitat suitability, change water velocities, depths, cover, and substrate preferred by fish, disrupt transport of organic matter and sediment, obstruct fish access to spawning grounds, decrease fish density and richness, limit instream species' access to feeding and refuge zones, and increase fish energetic swimming costs^1,3–6^.

Restoring degraded streams by enhancing habitat complexity or heterogeneity has been one of the primary objectives of river restoration projects in recent decades^7,8^. Instream habitat complexity can be divided into hydraulic complexity, which may include spatial and temporal variations in local flow characteristics such as depth, velocity, and turbulence, and structural complexity such as spatial variability of shade, cover, vegetation, substrate, and woody debris in streams^8–10^. Both types of complexities may substantially benefit instream habitats^9,11^. Enhanced complexity in a stream may enhance the biotic diversity of macroinvertebrates as well as fish, and also may increase the availability of favorable habitats for spawning, foraging, and refuge^7–9,12,13^. The instream hydraulic complexity may be evaluated through hydraulic characteristics such as flow depth and velocity variations, velocity gradients, vortices, and turbulence parameters^9,14,15^. Variations in hydraulic characteristics may restrict the movements of individual fish or groups of species due to physiological limits to swimming speeds and endurance^16^. Specifically, velocity gradients may be a major factor in the selection of habitats and feeding locations of fish species such as brown trout (*Salmo trutta*), and steelhead (*Oncorhynchus mykiss*)^17,18^. Moreover, in complex, turbulent flow fields, which are common in natural habitats, fish may utilize velocity magnitude and gradients, as well as turbulence characteristics to make directed movements^19^ and seek refuge^20^. Vortices in a stream may also facilitate feeding on drifting material, detecting prey for piscivorous predators, and fish swimming performance^14,20–22^.

1-D models (e.g., PHABISM) or 2-D models (e.g., weighted usable area, WUA) have been conventionally used to evaluate instream habitat suitability^9,14,23^. However, these models have several limitations and mostly estimate habitat suitability based on reach-scale flow characteristics or local velocity, depth, and substrate, ignoring complex flow patterns around instream topographical features such as large roughness elements, which commonly exist in natural habitats^9,14,15,23^. They generally do not incorporate effects of flow turbulence characteristics, which may have a significant influence on instream habitat and fish swimming^16,23,24^. Therefore, while performing numerical modeling, or experimental/field measurements, capturing complex flow patterns offers a more accurate understating of habitat complexity, especially in topographically diverse reaches.

Many research studies have attempted to examine the habitat suitability (for example in terms of availability and quality) and stream complexity through quantification of stream physical properties such as depth, velocity, substrate, turbulence, and the available cover in combination with ecological factors such as fish density and richness, fish passage efficiency, and availability of spawning grounds^9,15,16,23,25,26^. A set of hydraulic metrics has been used to quantify flow recirculation and spatial changes in flow kinetic energy in a desired scale and direction^15,27^. It has been argued that these metrics may be indicators of the instream habitat complexity and are of potential biological and ecological importance^15,27^. Quantifying spatial changes in flow kinetic energy can provide an estimate of the required power for an organism to move between two points^27^. Flow recirculation relates to flow vorticity and is a measure quantifying the influence of existing eddies in a stream solely based on the eddies' strength regardless of their spinning direction^15^. Generally, these metrics can effectively describe spatially varying flow patterns near micro and macro topographic features such as river banks and instream structures^15,27^. Such habitat hydraulic complexity metrics were computed through numerical simulations and field measurements for reaches with and without isolated large boulders, and it was found that the complex flow patterns, created by boulders, could be effectively captured by these metrics^14,15,27^. Several field studies investigated habitat metrics mainly in areas with complex features such as confluences, bends, reefs, and boulders^9,11,25,26,28–31^. In addition, studies have related the habitat hydraulic complexity metrics to ecological factors such as the preferred habitat, available spawning grounds, and feeding locations^9,14,15,30,31^.

For constructed channels with structures mimicking natural features, enhanced habitat complexity and food availability over several years may result in fish growth and productivity close to natural^32,33^. The process may be left to evolve on its own over a longer time or expedited with built-in physical features mimicking sequences of river meanders, pool and riffles, or boulder gardens^1^. Adding nature-like elements such as rock or gravel riffles, large woody debris, and boulders to channelized streams is a widely employed technique that adds habitat complexity with morphological and flow changes at both reach- and patch-scale^1,2,32,34,35^. The aim is to improve heterogeneity, productivity, and connectivity to ecologically degraded streams by adding more suitable habitats and generating flow conditions that fish may prefer and navigate with less effort.

Boulder placement, either as individual elements or as a group with specific densities, can ecologically enhance instream habitat by changing the physical stream conditions, increasing water depth, creating velocity gradients and vortices, promoting local scour and deposition, and providing refuge zones and migratory paths for fish^3,12,36,37^. It has been reported that boulder placement in a rock-ramp or natural riffle arrangement can be effective based on factors such as fish passage and attraction efficiency, fish density and richness improvement, habitat productivity, and effective energy dissipation^32,33,36,38–40^.

Mean and turbulent flow characteristics have been extensively studied in the vicinity of boulders^36,37,41^; however, there is no study to systematically examine how the proposed habitat hydraulic complexity metrics^15,27^ vary due to different boulder placements. Our novel experimental study explicitly tested different rock-ramp boulder arrangements to (1) investigate the influence of boulder placement on the reach-averaged and local variations of the habitat hydraulic complexity metrics, (2) develop relationships (using dimensional analysis) between the habitat hydraulic complexity metrics and (a) reach-averaged flow characteristics, (b) relevant habitat ecological scales, and (c) boulder concentration to facilitate predictions, and (3) examine possible implications of the habitat hydraulic complexity metrics for habitat quality based on the previous findings in the literature and the experiments of this study using a flume at 1:1 scale. To the best of the authors’ knowledge, the above analyses have not been systematically covered in previous research studies. The findings of this research may improve restoration practitioners’ understanding to more efficiently and frequently incorporate habitat hydraulic complexity metrics to stream ecological restoration projects that employ the boulder placement technique. The findings also may provide a practical measure to more conveniently predict habitat hydraulic complexity metrics in reaches with boulders in a rock-ramp arrangement.

## Methods

### Flume description

**Figure 1:**
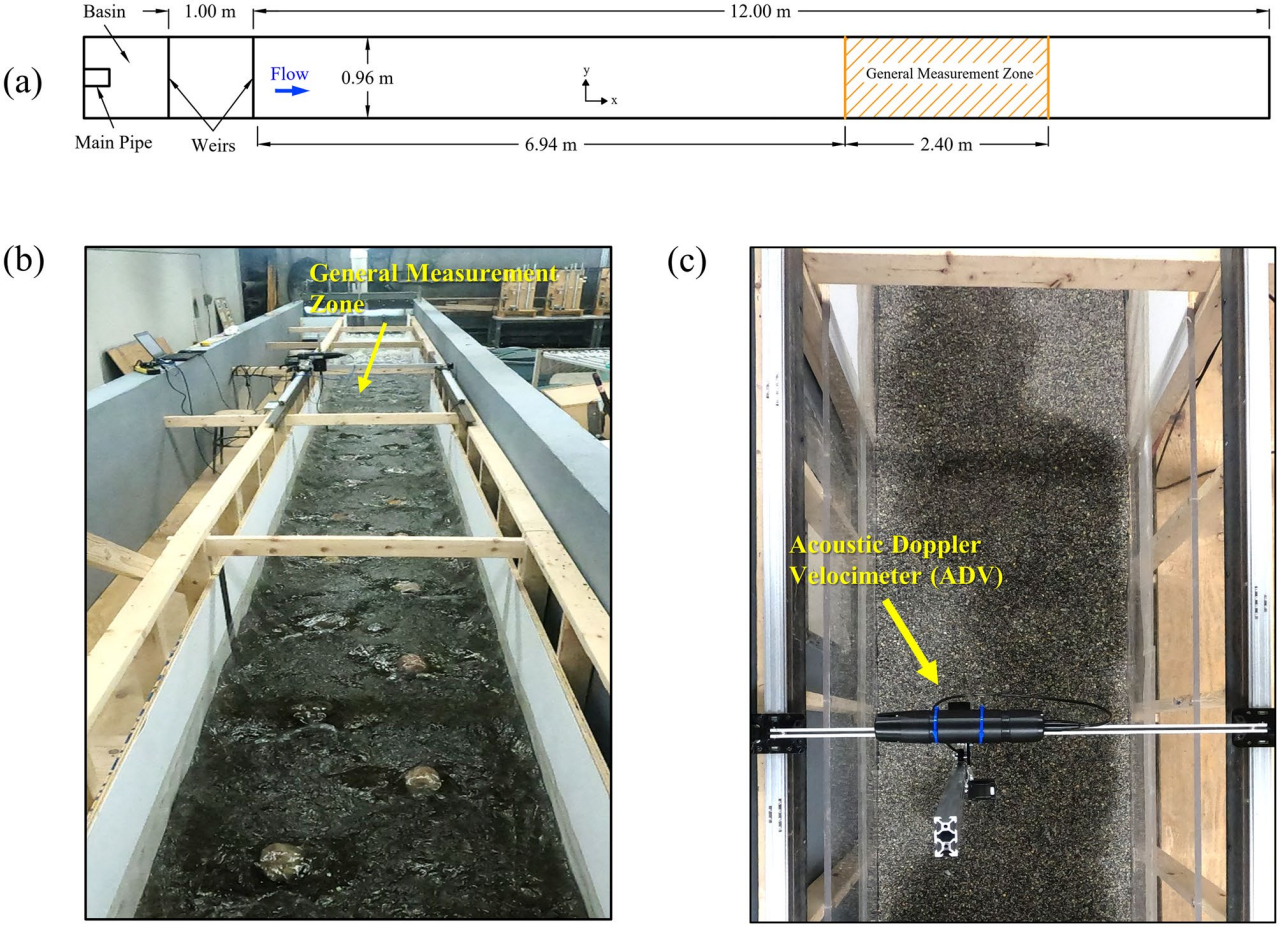
(**a**) A simplified plan view of the flume; (**b**) a view of the flume (facing upstream) during the experiments; (**c**) a plan view of the general measurement zone within the flume.

Experiments were conducted in the Ecohydraulics Flume located at Clarkson University. It is a water-recirculating flume, which is 13.00 m long, 0.96 m wide, and 1.00 m high. The water was pumped to a basin and then entered the flume. At the beginning of the flume, two rectangular weirs were installed to provide a smooth entrance of flow to the flume. The Cartesian coordinate system was used where *x*, *y*, and *z* indicate the longitudinal, transverse, and vertical directions, respectively. Following the right-hand rule, the positive directions for *x*, *y*, and *z* are streamwise, from right to the left bank (facing toward downstream), and pointing outward from the bed, respectively. A general measurement zone was defined as a portion of the flume that was 2.40 m long starting 6.94 m downstream of the flume entrance, i.e., the second weir. Based on the preliminary measurements in this zone, the average pattern of water surface fluctuations remained constant indicating a hydraulically fully developed turbulent flow^37^. Figure 1 shows several views of the flume highlighting its important described features. The flume bed was covered with a 50-mm layer of fine gravel with d_10_ = 3.8 mm, d_50_ = 6.1 mm, and d_90_ = 8.8 mm, where *d*_*p*_ is the particle size for which *p*% of the particles are finer. As this study is not focusing on flow variations over a movable bed, the gravel layer was fixed to the bed to prevent possible sediment erosion and deposition, specifically near the boulders. The bed slope was set at *S*_*0*_ = 0.005 representing low-gradient streams^42^ which normally provide high-quality habitat and have been recommended for restoration using instream structures^42^. Bed levels and water surface elevations were measured in the general measurement zone over a grid with 50 mm spacing in both *x* and *y* directions. The average of bed level variations was considered as the mean bed level (virtual bed level), where *z* = 0. Therefore, *z* is defined as the vertical distance from the mean bed level. By subtracting the mean bed level from the measured water surface elevations, the flow depth for each point was obtained and the reach-averaged flow depth, *H*, was calculated by taking the average of the flow depths over the measurement grid. A down-looking acoustic Doppler velocimeter (ADV) was used to perform velocity time-series measurements. The ADV was mounted on a carriage in the general measurement area, which enabled the automatic movement of the ADV in three dimensions with a resolution of 0.1 mm (Fig. 1c).

### Experimental scenarios

**Figure 2:**
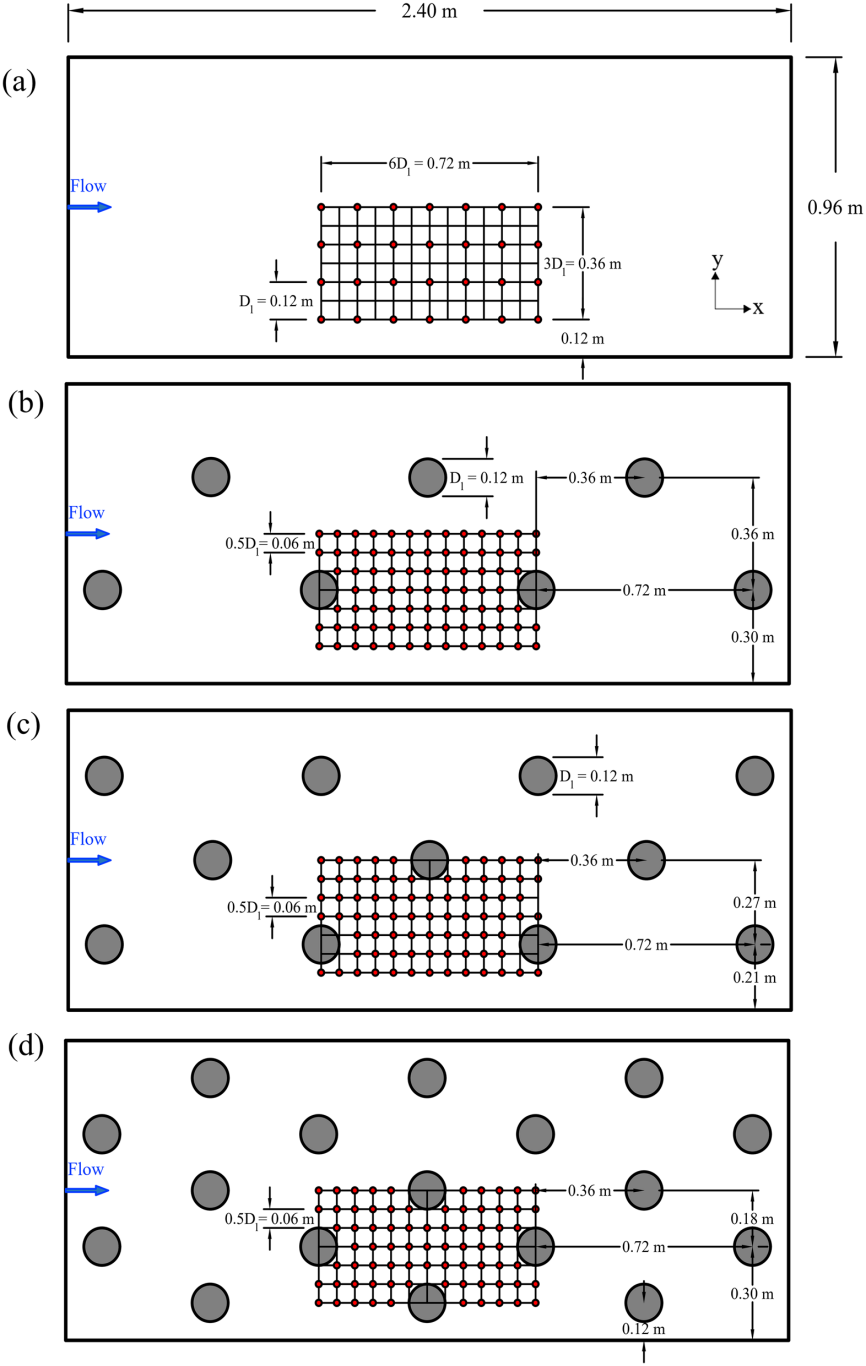
Scheme of the general measurement zone for experimental scenarios (**a**) S1-L and S1-H (no boulder scenarios), (**b**) S2-L and S2-H (λ = 3.4%), (**c**) S3-L and S3-H (λ = 5.4%), (**d**) S4-L and S4-H (λ = 8.3%). The grid shows the detailed measurement zone and orange circles show the location of measuring stations.

In total, eight experimental scenarios were conducted, including six scenarios with boulder placement and two scenarios without boulders as the reference scenarios. For scenarios including boulders, natural spherical-shape boulders with an equivalent size of *D*_*l*_ = 0.12 m in length and width, and *D*_*h*_ = 0.10 m in height were used. Several research studies have referred to large roughness elements with a similar size as boulders^36,41,43–45^. Boulders have also been referred to as large roughness elements that are not transported by flow^45^. However, it should be noted that following geological classifications, for the flume scale (1:1), the diameter of large roughness elements in this study falls in the range of cobbles (64 to 256 mm) rather than boulders (> 256 mm)^46^. To keep consistency with the literature, we refer to large roughness elements in this study as boulders, as well. Additionally, by applying the results to larger scales (e.g., for larger rivers and streams), the large roughness elements in this work may be classified as boulders^46^. A combination of two different flow rates (*Q*) and four boulder concentrations (*λ*) was used to design eight experimental scenarios. Two flow rates of *Q* = 0.060 and 0.075 m^3^/s were used to represent the low and high flow conditions. The selection of the minimum flow rate was restricted by the required minimum depth for the ADV measurements, and the high flow rate was selected in a way to minimize the possibility of bed abrupt detachment and boulder displacement during the experiments. Four boulder concentrations, *λ* = 0 (no boulder), 3.4, 5.4, and 8.3% were used to cover a variety of boulder concentrations. Figure 2 illustrates the boulder arrangements for the experimental scenarios and Table 1 summarizes experimental parameters. The boulders were placed in a rock-ramp (staggered) arrangement throughout the flume. While the longitudinal boulder-to-boulder spacing (*s*_*x*_) remained constant at *s*_*x*_ = 6*D*_*l*_, the transverse boulder-to-boulder spacing (*s*_*y*_) and the number of rows (*n*_*r*_) varied to create different boulder concentrations. For *λ* = 3.4, 5.4 and 8.3%, the varying parameters were *s*_*y*_ = 3*D*_*l*_, 2.25*D*_*l*_, and 1.5*D*_*l*_, and *n*_*r*_ = 2, 3, and 5, respectively. The resulting boulder concentrations generated isolated-roughness (for *λ* = 3.4%), and wake-interference flow regimes (for *λ* = 5.4% and 8.3%)^44,47^. In isolated-roughness flow regimes, boulders act independently, which means downstream wakes and vortices are completely developed and dissipated before the next boulder and there is no interaction between wakes of the boulders^47^. In wake-interference flow regimes, vortex generation and wake zones are not fully developed and dissipated before the next element, and interference between wakes of the boulders can be observed^47^. For the experimental scenarios, the boulder submergence ratios varied in the range of *H/D*_*h*_ ≈ 1.0–1.5, resulting in an intermediate submergence ratio^48^. Following a more detailed classification^49^, the submergence ratios in this range can be classified into Regime 3 (*H/D*_*h*_ ≈ 1.1–1.3; Froude number range ≈ 0.29 to 0.48) and Regime 2 (*H/D*_*h*_ ≈ 1.3–4.0; Froude number range ≈ 0.34 to 0.56), only for scenarios S3-H and S4-H. In Regime 2, surface waves start to appear and the length of the recirculation zone is reported to be less than 1D with a strong backward flow near the bed^49^. In Regime 3, the obstacle influence is pronounced as evidenced by a rough water surface with two strong surface waves of short wavelength, while the free shear layer from the boulders causes mixing over the entire depth indicating a strong downwash flow. In this regime, some backward flow is also observed at the water surface as separated flows from the top and sides of the hemisphere combined to form a strong arch vortex^49^. Additionally, the width of the wake region in Regime 2 is reported to be almost equal to the roughness element diameter while in Regime 3 the corresponding width is about twice the size of the boulder^49^.

**Table 1:** Description of the experimental scenarios.

Scenario code	Flow rate, Q (m^3^/s)	Boulder concentration, λ (%)	Reach-averaged flow depth, H (m)	Reach-averaged velocity, U_reach_ (m/s)	Boulder submergence ratio, H/D_h_	Froude number, Fr	Reynolds number, Re
S1-L	0.060	0	0.076	0.61	N/A	0.70	52,100
S1-H	0.075	0	0.096	0.72	N/A	0.74	78,047
S2-L	0.060	3.4	0.100	0.47	1	0.47	52,225
S2-H	0.075	3.4	0.127	0.54	1.3	0.49	78,018
S3-L	0.060	5.4	0.115	0.44	1.1	0.42	57,125
S3-H	0.075	5.4	0.141	0.55	1.4	0.47	87,358
S4-L	0.060	8.3	0.129	0.43	1.3	0.38	61,861
S4-H	0.075	8.3	0.151	0.50	1.5	0.42	86,123

To take velocity measurements, as shown in Fig. 2, a 0.72 × 0.36 m fragment within the general measurement zone was selected as the detailed measurement zone. The detailed measurement zone can be representative of the flow characteristics in the entire flume based on symmetry. The edge of the detailed measurement zone was located at least 0.12 m away from the flume sidewall to minimize the wall effects on the measurements^41^. The velocity measurements were taken in the detailed measurement zone over a grid with a longitudinal and transverse spacing of *D*_*l*_/2 = 0.06 m. Each grid point was chosen as a measuring station. At each measuring station, the velocity time series at three points with approximate relative depths of *z/H* = 0.05, 0.20, and 0.40 were recorded over the flow depth. Due to the ADV limitations in shallow depths, it was not possible to take measurements at higher relative depths. For scenarios without boulders (S1-L and S1-H), due to insignificant flow variability, a coarser grid spacing with a longitudinal and transverse spacing of *D*_*l*_ = 0.12 m was selected, and at each station, the velocity time-series were recorded only at *z/H* = 0.05 and 0.40. In these two scenarios, the skipped measuring points were interpolated (moving average method) to create a grid consistent with the spacing in other scenarios. At a few stations, due to extreme proximity to the boulders, the ADV receivers interfered with the boulders and no measurements were taken. These blocked points were interpolated (moving average method) as well to fill the measurement grid.

The reach-averaged velocity magnitude, *U*_*reach*_, for each scenario was calculated by averaging the measured velocity magnitudes in the detailed measurement zone at *z/H* = 0.40, where $U = \sqrt {\bar{u}^{2} + \bar{v}^{2} + \bar{w}^{2} }$ is the velocity magnitude, and $\bar{u}$, $\bar{v}$, and $\bar{w}$, are time-averaged streamwise, transverse, and vertical velocities, respectively. The Froude number (*Fr*) was in the range of 0.38 < *Fr* < 0.70, and the Reynolds number (*Re*) was in the range of 52,100 < *Re* < 87,358, indicating a fully turbulent flow (*Re* > 10^4^) in all the scenarios. Here, ${Fr} = {{U}}_{{reach}}/\sqrt{{gH}}$ and $Re= \rho{{U}_{reach}}{H/\mu}$, where *g* is the gravitational acceleration, *ρ* is water density, and *μ* is the dynamic viscosity of water.

### Data collection and treatment

Velocity time-series were recorded by deploying a down-looking Vectrino Plus (Nortek) ADV. The Vectrino Plus ADV samples the data in a cylindrical measurement volume located approximately 0.05 m below the probe; therefore, it is not possible to take measurements approximately in the upper 0.05 m of the flow depth. Data were sampled at a frequency of *f* = 100 Hz. Although the Vectrino Plus can sample at frequencies up to 200 Hz, initial assessments revealed that sampling at frequencies more than 100 Hz resulted in higher noise levels. The measurement duration was *T* = 120 s, which is long enough to accurately provide information on the mean and turbulent flow parameters^50^. To minimize the near-bed effects, for points at *z/H* = 0.05, the measurement volume height was adjusted to 2.5 mm^50^ while for the other points, it was 4.0 mm. To despike the recorded velocity time-series, phase-space threshold algorithm^51,52^ was applied. Afterward, signal correlation (COR) and signal-to-noise ratio (SNR) thresholds were applied to filter out poor-quality data. Removal of data points with COR ≤ 70% and SNR ≤ 15 dB conventionally has been used to improve data quality; however, a low COR value is not essentially a symptom of low-quality data^53^, specifically in the areas with higher velocity gradients such as near the stream beds and wakes of the boulders^54^. In this study, for each measured point, COR ≤ 70% and SNR ≤ 15 dB were initially applied to the data; in the case of less than 70% data remaining after applying these criteria, the COR threshold gradually (5% increments) decreased to a minimum of 40% until at least 70% of data were retained^50,55^. Removed data points were not replaced.

### Habitat hydraulic complexity metrics

To examine the flow complexity and influences of the boulder placement on the instream habitat availability and quality, three habitat hydraulic complexity metrics, *M*_*1*,_ *M*_*2*_, and *M*_*4*_, were used^15,27^. *M*_*1*_ shows the spatial gradient of kinetic energy between two points per unit mass and unit length:

1 $${M_{1} = U_{{avg}} \left| {\frac{{(U_{2} - U_{1} )}}{{\Delta s}}} \right|}$$

where *U*_*1*_ and *U*_*2*_ are velocity magnitudes at points 1 and 2, *U*_*avg*_ is the average velocity magnitudes of points 1 and 2, and Δ*s* is the spacing between points 1 and 2 in the desired direction. This metric is proportional to the acting drag force on an organism and can be used to estimate the expended power by an organism to move between two points^27^. The metric *M*_*2*_ can be defined as:

2 $${M_{2} = ~2U_{{avg}} \frac{{\left| {\frac{{(U_{2} - U_{1} )}}{{\Delta s}}} \right|}}{{U_{{\min }}^{2} }}}$$

where *U*_*min*_ is the minimum velocity between points 1 and 2. This metric is a scaled version of *M*_*1*_, which is divided by the flow kinetic energy at the point with the lower velocity. *M*_*2*_ indicates the average rate of change in kinetic energy per unit mass and unit length between two points^11^. It also can be considered as a measure of the required energy for an organism to move between two points^27^. In this study, *M*_*1*_ and *M*_*2*_ were calculated in the streamwise direction between two points, i.e., Δ*s* = Δ*x*. Both *M*_*1*_ and *M*_*2*_ are calculated at a single point. The metric *M*_*4*_ shows the modified recirculation in the horizontal plane, i.e., around the *z*-axis. It can be interpreted as the required energy by an organism to maintain its position without spinning around the *z*-axis^28^. Unlike the two other metrics, *M*_*4*_ is calculated for an area rather than a point. For a selected zone, the zone can be divided into cells and an *M*_*4*_ component, *M*_*4, com*_, is computed for each cell. Then, *M*_*4*_ for the selected zone can be found from the summation of calculated *M*_*4, com*_ for each cell:

3 $${M_{4}} = \sum M_{{4,~com}} = \sum \frac{{\left| {\xi _{z} } \right|\Delta A}}{{A_{{tot}} }} = \frac{{\sum \left| {\frac{{\Delta \bar{v}}}{{\Delta x}} - \frac{{\Delta \bar{u}}}{{\Delta y}}} \right|\Delta x \cdot \Delta y}}{{\sum \Delta x \cdot \Delta y}}$$

where ${\xi }_{z}$ is the vorticity around the *z*-axis at a point, Δ*A* is the area for each cell, and *A*_*tot*_ is the area of the desired zone. The advantage of using a modified recirculation metric over a regular recirculation metric (i.e., ${{\Sigma \xi _{z} \Delta A} / {A_{{tot}} }}$) is that in the regions with both positive and negative vorticity, the vorticities will not be canceled out by each other, so the modified recirculation will be a better indicator of the flow complexity in a region^15^. Here, to calculate *M*_*4*_, the detailed measurement zone was divided into square cells, i.e., Δ*x* = Δ*y*. It should be noted that *M*_*3*_ is similar to *M*_*4*_ but indicates the modified recirculation in the vertical plane transverse to the flow. In this study, due to the shallow flow depth in the scenarios, and subsequently small spacing between vertical points, only *M*_*4*_ was studied.

The habitat hydraulic complexity metrics were calculated using depth-averaged velocities at each measuring station within the detailed measurement zone. The Δ*s* values in an investigation should be ecologically meaningful, by selecting a characteristic size of the length size of an organism, the required travel distance for an organism to obtain food, or the size of roughness elements such as boulders^11^. For example, fish length would be an ecologically meaningful scale for Δs since fish speed, endurance and distance travelled generally increase with fish length^16^. Based on its ecological significance, the grid spacing can vary from 1 mm to a few meters^29^. Previous studies have used Δ*s* values in a wide range of 0.1–25.0 m to compute the habitat hydraulic complexity metrics^9,11,27,29^. Generally, in field studies, the selection of small Δ*s* requires the collection of a large amount of data; however, in laboratory and numerical studies, the selection of smaller Δ*s* is more feasible. In this paper, a spacing of Δ*s* = 0.06 = *D*_*l*_/2, which is proportional to the boulder size, was used to compute the metrics as a default spacing (Δ*x* = 0.06 m for *M*_*1*_ and *M*_*2*_ and Δ*x* = Δ*y* = 0.06 m for *M*_*4*_). The effects of grid size on the habitat hydraulic complexity metrics will be discussed later in the paper.

### Statistical analysis

In assessing whether the effect of changing a parameter such as flow rate, boulder concentration, and grid size, on the habitat hydraulic complexity metrics was significant, the *p-*values from a two-sided t-test were reported. The number of samples (n) for the t-test was the number of points or cells over which the metrics were computed in each scenario, varying between 65 to 91. Since the number of samples in all the cases exceeded 25, a t-test can be used regardless of the normality of the data^56^. The significance threshold was set at α = 0.05.

### Dimensional analysis

Calculation of the habitat hydraulic complexity metrics generally requires collecting velocity data over a 2D or 3D grid in the desired area. Collecting this amount of data is usually difficult, especially in the field as well as in situations that require smaller grid spacing. Therefore, to assess the complexity of a stream, it would be practical to estimate average metrics from flow characteristics and channel properties. A dimensional analysis was performed to find the possible relationships between habitat hydraulic complexity metrics and reach-averaged flow characteristics in the reaches with boulders. The metrics were predicted to be dependent on *H*, *U*_*reach*_, *D*, *Δs*, *Q*, *ρ*, *g*, *μ,* and *λ*. For *M*_*1*_, this relationship can be expressed as below:

4 $${\phi _{1} \left( {M_{1} ,~H,~U_{{reach}} ,~D,~\Delta s,~Q,~\rho ,~g,~\mu ,~\lambda } \right) = 0}$$

where $\phi$ is a functional symbol. Following the Buckingham-Π theorem^57^ and taking *ρ*, *U*_*reach*_, and *H* as the repeating variables the following dimensionless terms can be obtained:

5 $${\phi _{2} \left( {\frac{{M_{1} H}}{{U_{{reach}} {^{2}} }},~\frac{Q}{{U_{{reach}} H^{2} }},\frac{D}{H},\frac{H}{{\Delta s}},~\text{Re} ,~Fr,~\lambda } \right) = 0}$$

In all the scenarios in this study, the flow is fully turbulent and the viscous effects can be neglected. As a result, the Reynolds number can be eliminated from Eq. (5). Assuming *Q ≈* ${{U}}_{{reach}}$ *BH* (*B* is the flume width), then $Q/{{U}}_{{reach}}{{{H}}}^{2}$ *≈ B/H*, which implicitly takes into account the term *D/H* (*D* is constant in this study). Previous studies also found a strong relationship between flow rate and *D/H* in reaches with boulders^36^. Therefore, *D/H* can also be eliminated from Eq. (5). Then, Eq. (5) will be simplified as follows:

6 $${\frac{{M_{1} H}}{{U_{{reach}} {^{2}} }} = \phi _{3} \left( {\frac{Q}{{U_{{reach}} H^{2} }},~\frac{H}{{\Delta s}},~Fr,~~\lambda } \right)}$$

Similarly, the following expressions can be obtained for the metrics *M*_*2*_ and *M*_*4*_:

7 $${M_{2} H~ = ~\phi _{5} \left( {\frac{Q}{{U_{{reach}} H^{2} }},~\frac{H}{{\Delta s}},~Fr,~\lambda } \right)}$$

8 $${\frac{{M_{4} H}}{{U_{{reach}} }} = ~\phi _{6} \left( {\frac{Q}{{U_{{reach}} H^{2} }},~\frac{H}{{\Delta s}},~Fr,~~\lambda } \right)}$$

## Results and discussion

### Effects of grid spacing on habitat hydraulic complexity metrics

**Figure 3:**
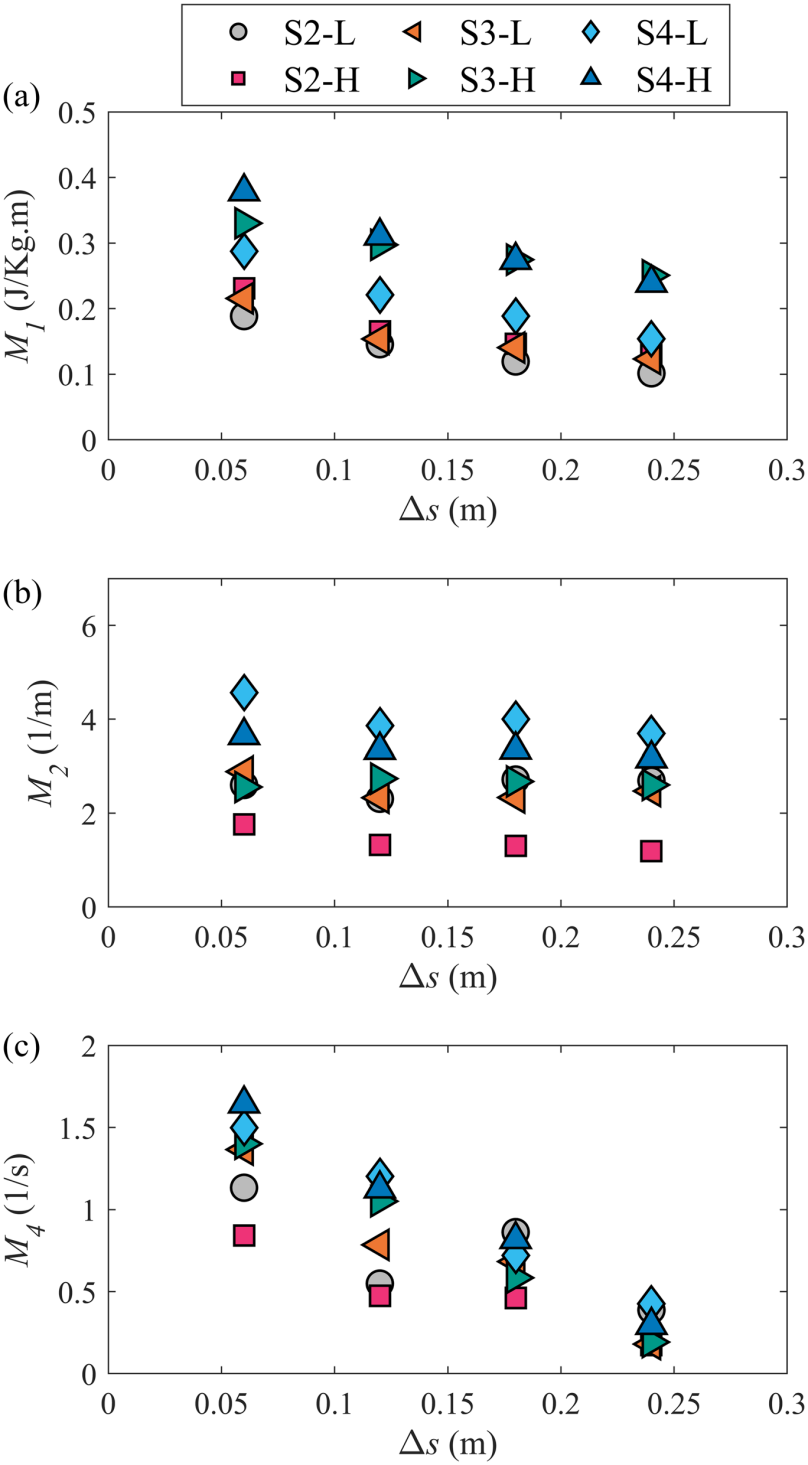
Variation of the habitat hydraulic complexity metrics with grid spacing (Δ*s*) for scenarios with boulder placement. (**a**) kinetic energy gradient metric, *M*_*1*_, (**b**) normalized kinetic energy gradient metric, *M*_*2*_, (**c**) modified recirculation metric *M*_*4*_*.*

The sensitivity of the habitat hydraulic complexity metrics to Δ*s* was examined by calculating the metrics for Δ*s* = 0.06, 0.12, 0.18, and 0.24 m (for *M*_*4*_, Δ*s* = Δ*x* = Δ*y*). Figure 3 shows the variation of the metrics with grid spacing for scenarios with boulders. A preliminary assessment of no-boulder scenarios (S1-L and S1-H) showed that all the metrics decreased by increasing the grid spacing. However, because the metrics are mostly used in complex rather than non-obstructed and 1-D flows, the plots only include scenarios with boulder placement to highlight the effects of grid spacing on the metrics in complex flows. All the metrics generally decreased as Δ*s* increased. At the low flow rate, by changing the Δ*s* from the smallest to largest, i.e., 0.06 m to 0.024, the mean decreases in the *M*_*1*_, *M*_*2*_, and *M*_*4*_ metrics (averaged over all the scenarios with boulders) were 45.1, 9.9, and 74.7%, respectively. At the high flow rate, these reductions were 34.8, 14.7, and 82.5% for *M*_*1*_, *M*_*2*_, and *M*_*4*_, respectively. Table 2 shows the *p*-values associated with the changes in the metrics due to increasing Δ*s* from 0.06 to 0.24 m for all scenarios. The table indicates that changes in *M*_*1*_ and *M*_*4*_ were statistically significant while for *M*_*2*_ they were not (*p*-values > 0.05 for all scenarios except for S2-H). This result indicated the considerable influence of grid spacing on *M*_*1*_ and *M*_*4*_ metrics in the reaches with boulder placement. Additionally, the differences in the reported average reductions due to changing the flow rate were less than 10%, indicating an insubstantial effect of flow rate on the habitat hydraulic complexity metrics' sensitivity to the grid spacing. The significant sensitivity of the metrics *M*_*1*_ and *M*_*4*_ to the grid spacing in this study is contrary to the findings of a previous study in which an insignificant correlation was found between the habitat hydraulic complexity metrics and Δ*s*^29^. This difference can be attributed to different topographic features in the studied reaches. In the previous findings, measurements were mainly taken around the bends and reaches with no significant obstruction^29^, in which a more uniform flow with smaller velocity gradients is expected. However, in this study, the systematic boulder placement generated more complex flow patterns with noticeable velocity gradients. Therefore, due to the variations of flow velocities in the zone studied, substantially different values for the metrics are anticipated by computing the metrics over different spatial scales.

**Table 2:** p-values associated with changing the grid spacing from 0.06 to 0.24 m.

Scenario code	*p* value		
	*M*_*1*_ (J/kg m)	*M*_*2*_ (1/m)	*M*_*4*_ (1/s)
S2-L	< 0.001*	0.463	0.001*
S3-L	< 0.001*	0.251	< 0.001*
S4-L	< 0.001*	0.129	< 0.001*
S2-H	< 0.001*	0.004*	< 0.001*
S3-H	0.011*	0.435	< 0.001*
S4-H	< 0.001*	0.165	< 0.001*

*M*_*1*_ is the kinetic energy gradient metric, *M*_*2*_ is the normalized kinetic energy gradient metric, and *M*_*4*_ is the modified recirculation metric. (*) indicates the change is statistically significant assuming a significance level of 0.05.

### Statistical distribution of habitat hydraulic complexity metrics

Table 3 lists the mean, minimum, maximum, and standard deviations of the habitat hydraulic complexity metrics (Δ*s* = 0.06 m*)* for all the scenarios. To complement the results from Table 3 and assess whether the influences of solely changing the boulder concentration or flow rate on the metrics were statistically significant, Table 4 shows *p*-values associated with changing flow rate from low to high for a given boulder concentration, and Table 5 shows *p*-values associated with gradually increasing the boulder concentration for a given flow rate.

**Table 3:** The statistical parameters of the habitat hydraulic complexity metrics in the detailed measurement zone.

Habitat hydraulic complexity metric	*M*_*1*_ (J/kg m)				*M*_*2*_ (1/m)				*M*_*4*_ (1/s)
Scenario code	Mean	Min	Max	SD	Mean	Min	Max	SD	Mean
S1-L	0.14	0.001	1.15	0.22	0.82	0.005	8.82	1.44	0.21
S1-H	0.20	0.0002	1.45	0.30	0.84	0.001	7.29	1.35	0.20
S2-L	0.19	0.001	0.99	0.18	2.60	0.01	52.61	5.98	1.13
S2-H	0.22	0.02	0.95	0.22	1.73	0.11	17.38	2.26	0.84
S3-L	0.21	0.006	1.04	0.20	2.88	0.06	31.06	4.12	1.36
S3-H	0.33	0.003	1.60	0.30	2.56	0.02	12.43	2.37	1.40
S4-L	0.29	0.004	1.15	0.22	4.56	0.07	46.95	6.70	1.49
S4-H	0.38	0.003	1.22	0.28	3.65	0.04	28.03	3.96	1.64

*M*_*1*_ is the kinetic energy gradient metric, *M*_*2*_ is the normalized kinetic energy gradient metric, and *M*_*4*_ is the modified recirculation metric.

*Min* minimum, *Max* maximum, *SD* standard deviation.

**Table 4:** p-values from a t-test associated with changes in flow rate for a given boulder concentration.

Scenario code	*p-*value		
	*M*_*1*_ (J/kg m)	*M*_*2*_ (1/m)	*M*_*4*_ (1/s)
S2-L to S2-H	0.041*	< 0.001*	0.273
S3-L to S3-H	< 0.001*	0.106	0.269
S4-L to S4-H	0.002*	0.120	0.268

*M*_*1*_ is the kinetic energy gradient metric, *M*_*2*_ is the normalized kinetic energy gradient metric, and *M*_*4*_ is the modified recirculation metric. (*) indicates the change is statistically significant assuming a significance level of 0.05.

**Table 5:** p-values from a t-test associated with changes in boulder concertation for a given flow rate.

Scenario code	*p-*value		
	*M*_*1*_ (J/kg m)	*M*_*2*_ (1/m)	*M*_*4*_ (1/s)
S1-L to S2-L	0.001*	0.004*	< 0.001*
S2-L to S3-L	0.105	0.265	0.048*
S3-L to S4-L	0.002*	0.016*	0.174
S1-H to S2-H	0.135	< 0.001*	< 0.001*
S2-H to S3-H	0.001*	0.001*	< 0.001*
S3-H to S4-H	0.065	0.009*	0.015*

*M*_*1*_ is the kinetic energy gradient metric, *M*_*2*_ is the normalized kinetic energy gradient metric, and *M*_*4*_ is the modified recirculation metric. (*) indicates the change is statistically significant assuming a significance level of 0.05.

For metric *M*_*1*_, the mean *M*_*1*_ values for scenarios incorporating boulders showed the same order of magnitude as values from previous studies for reaches with single and multiple boulders^27^ but they were about one order of magnitude larger than calculated values in the confluence of two rivers^11^. Using a larger grid spacing in the study in the confluence of two rivers^11^ can be the reason for this difference. For a scenario at the higher flow rate, the mean *M*_*1*_ was on average (averaged for all the scenarios) 36% greater than its counterpart at the lower flow rate and this change in *M*_*1*_ values was statistically significant with *p* < 0.05 (Table 4). Adding boulders and subsequently increasing the boulder concentration resulted in a slight increase in the mean *M*_*1*_ values at both flow rates. This increase was statistically significant (*p* < 0.05) except for changes from S2-L to S3-L, S1-H to S2-H, and S3-H to S4-H with *p* = 0.105, 0.135, and 0.065, respectively (Table 5).

The minimum values of *M*_*1*_ did not show a specific trend with boulder concentration and flow rate. For a given boulder concentration, the maximum values of *M*_*1*_ were generally larger at the higher flow rate except for the lowest boulder concentration. Comparing the standard deviations revealed that for a given boulder concentration increasing the flow rate increased the variability of *M*_*1*_ as well. Furthermore, the standard deviations showed that for a given flow rate, increasing boulder concentration generally increased the variability of *M*_*1*_ values. The only exception was the change from the medium to the highest boulder concentration at the higher flow rate (S3-H to S4-H) where the standard deviation slightly decreased. For the lower and higher flow rates, the largest variability occurred at the highest boulder concentration (S4-L) and the medium boulder concentration (S3-H), respectively.

For metric *M*_*2*_, the mean *M*_*2*_ values for scenarios with boulders were of the same order of magnitude as those observed for a series of complex habitats, some including boulders^9,29^. According to another study, after the construction of several reefs in the river, the mean *M*_*2*_ increased due to variation in the local flow^25^. However, even after the reef construction, the calculated mean *M*_*2*_ values in their study^25^ were about two orders of magnitude smaller than the values estimated in this study. For a given boulder concentration, increasing the flow rate resulted in an average drop of 21% in the mean *M*_*2*_ values. This decrease was only significant (Table 4) for the lowest boulder concentration (S2-L to S2-H). Similar to *M*_*1*_, for a given flow rate, the mean *M*_*2*_ values gradually increased by increasing the boulder concentration. This increase was statistically significant for all scenarios except for the change from S2-L to S3-L (*p* = 0.265; Table 5).

Adding boulders (i.e., change from S1-L to S2-L as well as S1-H to S2-H) generally increased the minimum observed *M*_*2*_ values but similar to *M*_*1*_, minimum *M*_*2*_ values did not show a specific trend with boulder concentration and flow rate. The maximum *M*_*2*_ values were one to two orders of magnitude higher than the mean *M*_*2*_ values, indicating the presence of isolated large *M*_*2*_ values in the studied zone. They showed a similar order of magnitude with the calculated maximum *M*_*2*_ values in reaches with boulders in the previous findings^27^. The extremely large *M*_*2*_ values can be attributed to small values in the denominator of the *M*_*2*_ (Eq. 2) due to the significantly lower velocities in the wake of boulders^29^. This also can be associated with the drop in the mean *M*_*2*_ values after increasing the flow rate for a given boulder concentration. By increasing the flow rate, the low velocities in the wake of the boulders increased resulting in larger values in the denominator of the *M*_*2*_ equation and subsequently smaller *M*_*2*_ values. As expected, due to varying local flows, the variabilities of *M*_*2*_ values for scenarios with boulders were significantly higher. The largest standard deviations were for scenarios S2-L and S4-L, which were the same scenarios with the highest observed maximum *M*_*2*_ values.

For scenarios with boulders, the mean *M*_*4*_ values were one order of magnitude higher than the scenarios without boulders. The mean *M*_*4*_ values for scenarios with boulders showed the same order of magnitude as reported values in areas with vortices^14^, around boulders^15^, and in complex habitats^9^. However, the obtained values within the bends^29^ were about an order of magnitude smaller than the mean *M*_*4*_ values in this study. After adding boulders to the flume, a significant elevation in the mean *M*_*4*_ can be observed, specifically at the lower flow rate. After adding boulders, the mean *M*_*4*_ increased 438% and 320% for the lower and higher flow rates, respectively.

The changes in *M*_*4*_ values due to increasing the flow rate for a given boulder concentration did not follow a specific trend and were statistically insignificant (Table 4). Regardless of the flow rate, by gradually increasing the boulder concentration, the mean *M*_*4*_ also increased. These increases were statistically significant (Table 5) except for the change from S3-L to S4-L (*p* = 0.174; Table 5). The increase due to increasing boulder concentration was more noticeable at the higher flow rate compared with the lower flow rate. For instance, at the higher flow rate, by changing the boulder concentration from the lowest (S2-H) to the highest (S4-H), the increase in mean *M*_*4*_ was 95% while at the lower flow rate the same change in the boulder concentration (S2-L to S4-L) resulted in only 31% increase in mean *M*_*4*_.

### Spatial variations of habitat hydraulic complexity metrics

**Figure 4:**
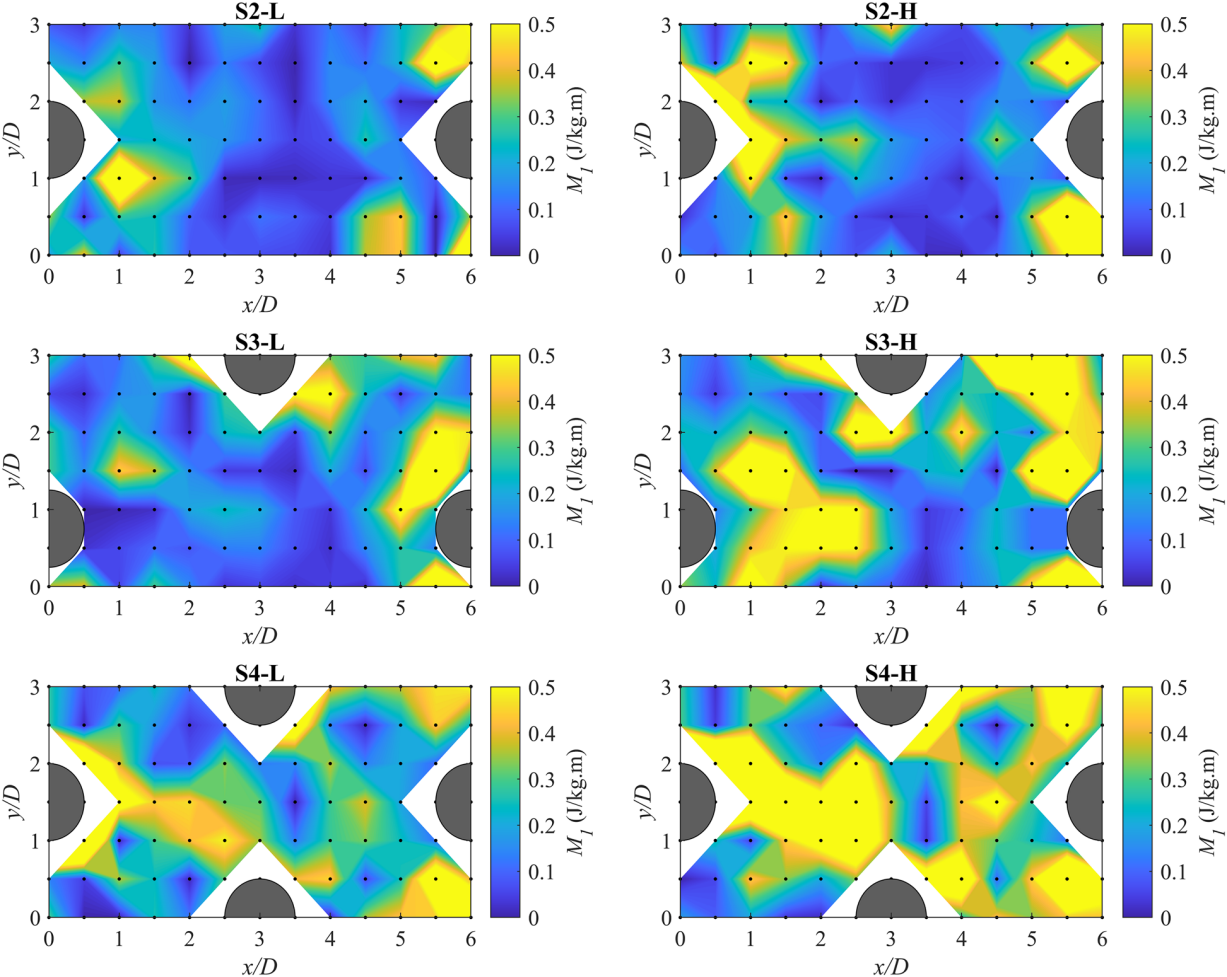
Contour maps of the kinetic energy gradient metric, *M*_*1*_, for scenarios with boulder placement in the detailed measurement zone for Δ*s* = Δ*x* = 0.06 m. Black dots show the measuring station locations. The blank (white) areas in the contour maps are due to the missing measuring points occupied by boulders.

To examine the spatial distribution of the habitat hydraulic complexity metrics in a reach with boulders, contour maps of the metrics *M*_*1*_ and *M*_*2*_ in the detailed measurement zone were generated. The metrics shown by contour maps were computed by Δ*s* = 0.06 m because the preliminary evaluation of the results showed that changing the grid spacing did not noticeably change the spatial distribution of the metrics as also reported by another study^11^. Figure 4 shows the contour maps of *M*_*1*_ for scenarios with boulders. High-*M*_*1*_ regions generally appeared downstream and at the sides of the boulders. At the higher flow rate, the high-*M*_*1*_ regions were more extended in comparison with the lower flow rate. For instance, while the high-*M*_*1*_ regions at the lower flow rate for scenarios S2-L, S3-L, and S4-L covered about 1*D*, 1*D*, and 2*D* downstream of the boulders, respectively, at the higher flow rate these regions extended to approximately 1.5*D*, 2.5*D*, and 3*D* downstream of the boulders. This can be attributed to the generally higher average velocities between two points (*U*_*avg*_) at the higher flow rate. At the lower flow rate, by increasing the boulder concentration from 3.4 to 5.4%, no substantial change in the spatial pattern of *M*_*1*_ values occurred; however, by further increasing the boulder concentration to 8.3%, high-*M*_*1*_ regions extended more noticeably. At the higher flow rate, the spatial patterns of *M*_*1*_ for the lowest boulder concentration (S2-H) differed from those patterns at the higher boulder concentrations (S3-H and S4-H) where high-*M*_*1*_ regions appeared more frequently over a larger area. These are compatible with the results from Table 3, in which the scenarios with the higher boulder concentration generally resulted in the larger variability (standard deviation) of *M*_*1*_ values in the detailed measurement zone.

**Figure 5:**
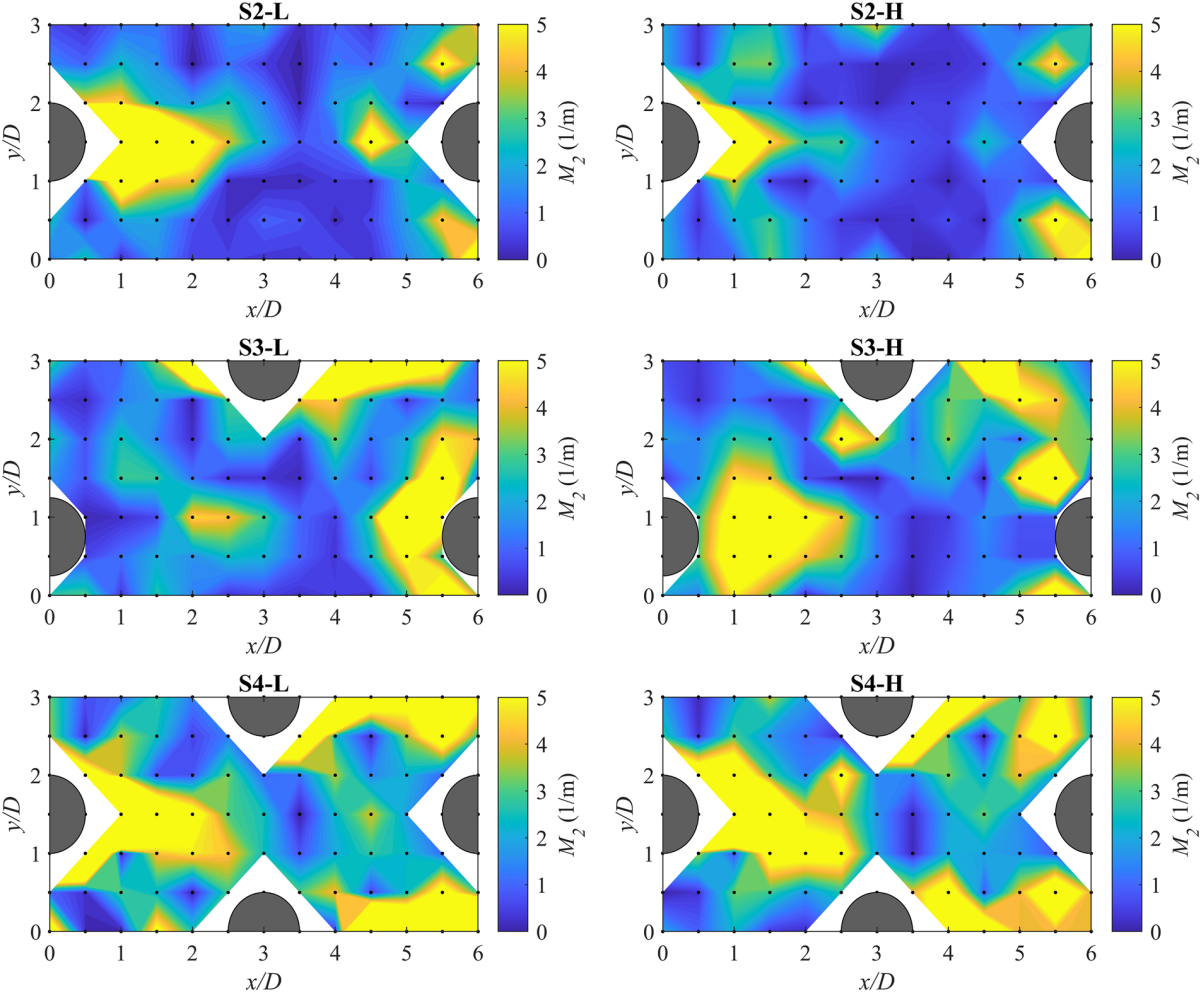
Contour maps of the normalized kinetic energy gradient metric, *M*_*2*_, for scenarios with boulder placement in the detailed measurement zone for Δ*s* = Δ*x* = 0.06 m. Black dots show the measuring station locations. The blank (white) areas in the contour maps are due to the missing measuring points occupied by boulders.

As shown in Fig. 5, similarly, high-*M*_*2*_ regions can be seen in the vicinity of the boulders, specifically in the wake of the boulders. For the smallest boulder concentration, λ = 3.4%, increasing the flow rate (i.e., change from S2-L to S2-H) reduced the extent of the high-*M*_*2*_ region in the boulder’s wake from 2.5*D* to 1.5*D*. For the medium boulder concentration, λ = 5.4%, by increasing the flow rate a high-*M*_*2*_ region appeared downstream of the leftmost boulder in the detailed measurement zone, and for the highest boulder concentration, λ = 8.3%, no specific change in the spatial patterns of *M*_*4*_ was observed as a result of increasing the flow rate. At both flow rates, increasing the boulder concentration generally resulted in the appearance of more high-*M*_*2*_ regions. The extremely large values of *M*_*2*_ occurred immediately downstream of the boulders, where extremely low (near-zero) velocities were observed in the recirculation zone of the boulders resulting in very small values in the *M*_*2*_ equation, as discussed in the previous section.

**Figure 6:**
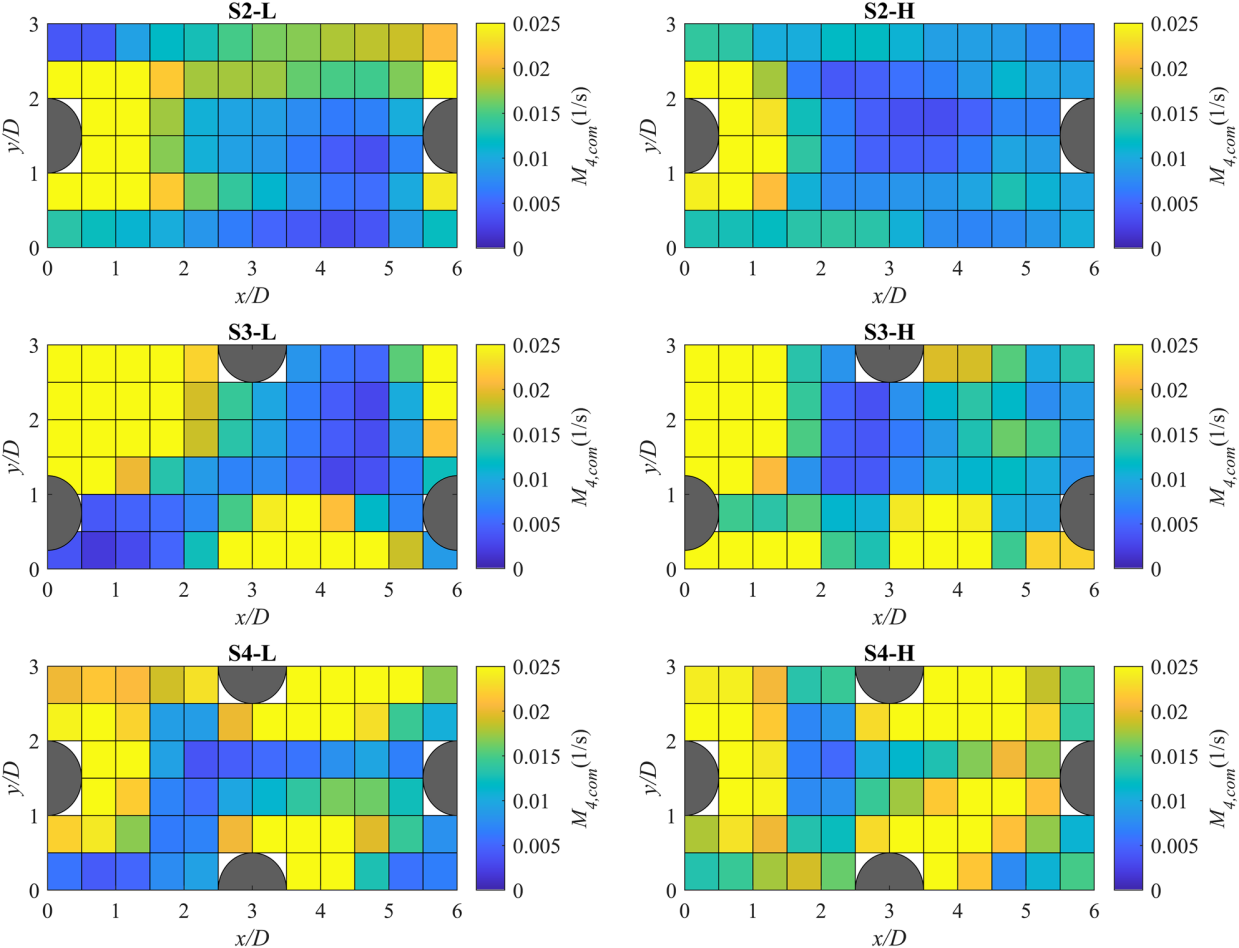
Contour maps of the components of modified recirculation metric, *M*_*4,com*_, for scenarios with boulder placement in the detailed measurement zone for Δ*s* = Δ*x* = Δ*y* = 0.06 m. The blank (white) areas in the contour maps are due to the missing measuring points occupied by boulders.

Figure 6 shows the contour map of ${{M}}_{{4, com}}$ in detailed measurement zone computed for cells with Δ*x* = Δ*y* = 0.06 m. It provides information about the local modified recirculation and the regions that highly contribute to the computed *M*_*4*_ for the entire reach. Generally, zones with higher ${{M}}_{{4, com}}$ were in the wake regions and at the sides of the boulders. For the scenario with λ = 3.4%, by increasing the flow rate, the extent of cells with higher ${{M}}_{{4, com}}$ in the wake of the boulders slightly decreased from about 2*D* to 1.5*D*. For λ = 5.4%, by increasing the flow rate, the extent of the region with higher local modified recirculation downstream of the leftmost boulder was reduced from 2.5*D* to 1.5*D*; however, a region with high ${{M}}_{{4, com}}$ appeared at the right side of that boulder. For λ = 8.3%, the number of cells with higher modified recirculation generally increased in-between boulders at both flow rates. Generally, by increasing the boulder concentration at both flow rates, the number of cells with higher ${{M}}_{{4, com}}$ increased. These findings corroborate that increasing the boulder concentration results in higher *M*_*4*_ values in the detailed measurement zone as seen in Table 3.

Previous findings also indicated increased metrics downstream, at sides, and in-between randomly placed boulders with different sizes and submergence ratios^14,15,27^. However, to the best of the authors’ knowledge, the spatial variation in habitat hydraulic complexity metrics due to systematic boulder placement has not been assessed in previous studies.

Although flows in two experimental scenarios (S3-H and S4-H) were classified in different flow regimes (Regime 2) than the other scenarios (Regime 3), no significant influence of submergence ratio on the spatial distribution of metrics was identified. This might be due to the fact that the submergence ratios of the experimental scenarios were relatively close and the influence of this factor on the flow field remained insignificant even though the flows in S3-H and S4-H were technically classified as a different flow regime.

### Estimation of habitat hydraulic complexity metrics

**Figure 7:**
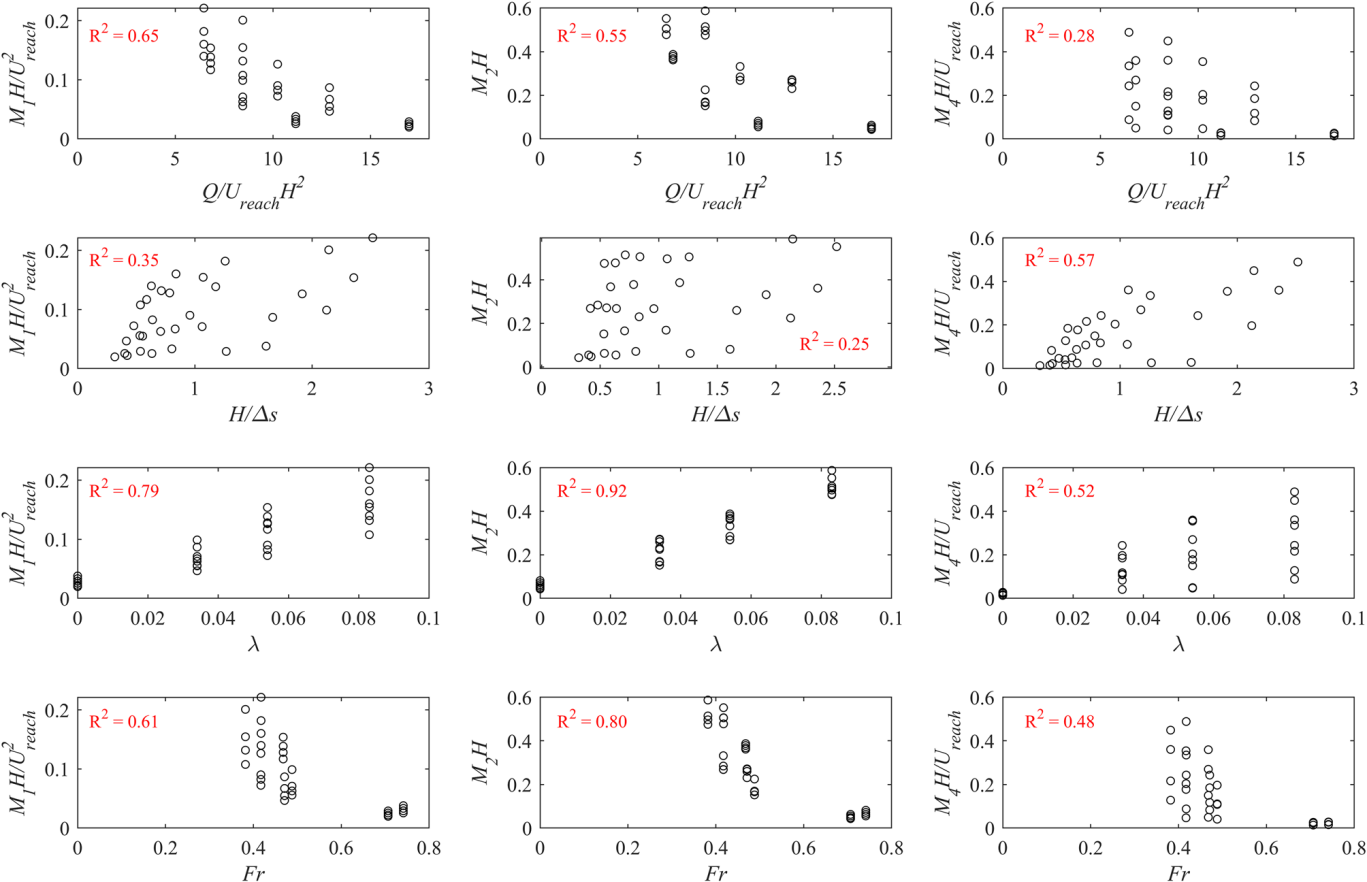
The relationships between the habitat hydraulic complexity metrics and derived dimensionless parameters from the dimensional analysis. *M*_*1*_ is the kinetic energy gradient metric, *M*_*2*_ is the normalized kinetic energy gradient metric, and *M*_*4*_ is the modified recirculation metric. *H* is reach-averaged flow depth, *U*_*reach*_ is reach-averaged flow velocity, *Q* is flow rate, Δs is grid spacing, λ is the boulder concentration, and *Fr* is Froude number. The shown R^2^ values on each plot indicate the coefficient of determination from the best fit.

To further investigate the dependence between the derived dimensionless terms, the relationships between the dimensionless metrics and other dimensionless parameters were examined. Figure 7 shows the variation of the dimensionless habitat hydraulic complexity metrics *M*_*1*_*H/*${{U}}_{{reach}}$^*2*^, *M*_*2*_*H*, and *M*_*4*_*H/*${{U}}_{{reach}}$ with the other dimensionless terms, *Q/*${{U}}_{{reach}}$* H*^*2*^, *H/Δs*, *λ*, and *Fr*. The coefficient of determination, *R*^*2*^, was found based on the best available fit between the parameters. It should be mentioned that the dimensionless metrics were computed for Δ*s* = 0.06, 0.12, 0.18, and 0.24 m (for *M*_*4*_, Δ*s* = Δ*x* = Δ*y*). A negative correlation between the dimensionless metrics and *Q/*${{U}}_{{reach}}$* H*^*2*^ can be seen; however, *R*^*2*^ values for *M*_*2*_*H*, and *M*_*4*_*H/*${{U}}_{{reach}}$ were 0.55 and 0.28, respectively, indicating a weak to moderate correlation^58^. By increasing *H*/Δ*s,* the dimensionless metrics also increased. The correlation coefficient for *M*_*1*_*H/*${{U}}_{{reach}}$^*2*^, and *M*_*2*_*H* were 0.35 and 0.25, respectively, which indicated weaker correlations with *H*/*Δs*. As λ increased, the dimensionless metrics also increased, and the correlation coefficients for *M*_*1*_*H/*${{U}}_{{reach}}$^*2*^, *M*_*2*_*H*, and *M*_*4*_*H/*${{U}}_{{reach}}$ were 0.79, 0.92, and 0.52, which indicated moderate to strong correlations between the dimensionless metrics and the boulder concentration. A negative correlation between the dimensionless metrics and *Fr* can be seen. The *R*^*2*^ values were 0.61, 0.80, and 0.48 for *M*_*1*_*H/*${{U}}_{{reach}}$^*2*^, *M*_*2*_*H*, and *M*_*4*_*H/*${{U}}_{{reach}}$, respectively, showing a moderate to a strong relationship between the parameters.

Although in some cases, especially for *M*_*2*_*H*, and *M*_*4*_*H/*${{U}}_{{reach}}$, weak to moderate correlations were observed; the derived dimensionless groups from the dimensional analysis can be used for estimating the habitat hydraulic complexity metrics. A multiple regression analysis was performed and the following expressions were found:

9 $${\frac{{M_{1} H}}{{U_{{reach}} {^{2}} }} = 16.55Fr^{{1.75}} \left( {\frac{Q}{{U_{{reach}} H^{2} }}} \right)^{{0.01}} \left( {\frac{H}{{\Delta S}}} \right)^{{0.33}} \lambda ^{{1.22}} }$$

10 $${M_{2} H = 8.32Fr^{{0.28}} \left( {\frac{Q}{{U_{{reach}} H^{2} }}} \right)^{{ - 0.02}} \left( {\frac{H}{{\Delta S}}} \right)^{{0.12}} \lambda ^{{1.00}} }$$

11 $${\frac{{M_{4} H}}{{U_{{reach}} }} = 1.12Fr^{{ - 0.22}} \left( {\frac{Q}{{U_{{reach}} H^{2} }}} \right)^{{0.16}} \left( {\frac{H}{{\Delta S}}} \right)^{{0.57}} \lambda ^{{0.74}} }$$

**Figure 8:**
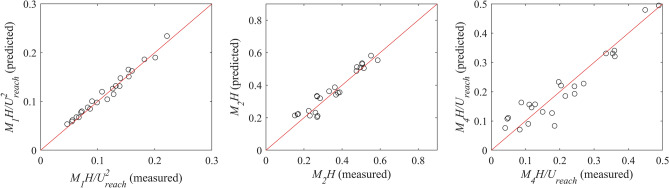
The predicted dimensionless habitat hydraulic complexity parameters from the proposed equations against the measured values from the experiments. *M*_*1*_ is the kinetic energy gradient metric, *M*_*2*_ is the normalized kinetic energy gradient metric, and *M*_*4*_ is the modified recirculation metric. *H* is reach-averaged flow depth, and *U*_*reach*_ is reach-averaged flow velocity. The red line shows *R*^2^ = 1.0.

By comparing the predicted values from Eqs. (9) to (11) and the measured values from the experiments (Fig. 8), the *R*^*2*^ values of *M*_*1*_*H/*${{U}}_{{reach}}$^*2*^*, M*_*2*_*H*, and *M*_*4*_*H/*${{U}}_{{reach}}$ for a 95% confidence level were 0.97, 0.91, and 0.88, respectively. These strong correlations showed acceptable performance of the proposed equations to predict the habitat hydraulic complexity metrics. Using Eqs. (9) to (11), the average habitat hydraulic complexity metrics for a reach with boulders can be estimated by only obtaining information about the reach-averaged depth and velocity, flow rate, boulder concentration (in rock-ramp arrangement), and the desired grid spacing.

However, the proposed equations were only based on the limited tested dataset and parameters and are only applicable to the investigated range of parameters in this study. It should be emphasized that in this study boulder concentration was calculated for boulders placed in a rock-ramp arrangement. One may obtain similar boulder concentrations for completely different arrangements such as boulder clusters, random (non-uniform) boulder placement, or even for an isolated boulder that covers a large area; however, the applicability of these relationships should be limited to rock-ramp arrangements due to significant changes in the local flow fields for the other configurations^59^. There are multiple other potentially influential factors, including but not limited to embedded depth, bed slope, and substrate composition that should be taken into account for designing a boulder placement scenario. Further numerical and experimental studies that incorporate these factors as well as assess the parameters investigated (e.g., boulder concentration, flow rate, etc.) over a broader range are needed to improve the accuracy and applicability of these equations in the field and under more complex conditions.

### Potential implications of habitat hydraulic complexity metrics for instream species

The habitat hydraulic complexity metrics may provide important information about the instream habitat quality and availability. Possible influences of habitat hydraulic complexity metrics, calculated over the default grid size (0.06 m) for the flume model (1:1 scale), on the instream habitat, as well as potential effects of increasing the grid size to the largest in this study (0.24 m) need further elaboration. As pointed out earlier, the spatial scale on which the metrics are calculated can noticeably influence the metric values. This makes comparing the metrics across the studies challenging as a variety of spatial scales have been used in different studies and sometimes scales are not incorporated. In the following comparisons, the spatial scales over which the metrics were computed were mostly larger or unreported and also were not necessarily based on an ecologically relevant scale. Even with these limitations, interpreting the metrics in this work in relation to habitat quality and availability for instream species may be helpful and provide insightful information for future work and projects.

Regions with noticeable longitudinal velocity and energy gradients as well as recirculation can be used to delineate suitable habitats such as spawning grounds in a reach^11,15,27^. Previous findings indicated a partial relationship between *M*_*1*_ values and spawn density per unit area of Chinese Sturgeons (*Acipenser sinensis*)^30^. It was found that the spawns were mainly observed in areas where *M*_*1*_ > 0.029 J kg^−1^ m^−1^. The *M*_*1*_ values in the investigated scenarios of this study exceeded 0.029 J kg^−1^ m^−1^ except for a small number of points between the boulders; however, as the boulder concentration and flow rate increased the frequency of regions with low *M*_*1*_ values reduced. Earlier, it was mentioned that increasing the grid size from 0.06 to 0.24 m resulted in an average reduction of *M*_*1*_ values up to 45.1%. Even by applying this reduction, most *M*_*1*_ values remained above 0.029 J kg^−1^.

Higher *M*_*2*_ values can be used to identify locations with noticeable biological richness and ideal feeding habitats^9,28^. It was predicted that *M*_*2*_ values in the range of 4–14 m^−1^ can be distinguished as a suitable location for brown trout feeding^14^. In this study, before placing boulders, the observed *M*_*2*_ values were mainly below this range but after adding boulders the regions with *M*_*2*_ values in the range of 4–14 m^−1^ appeared. These regions were located mainly in the middle of wake regions, i.e., about 1.5*D* downstream of the boulders. Increasing the boulder concentration, generally expanded regions with *M*_*2*_ values in this specific range. The extremely large *M*_*2*_ values in the near-wake region did not fall in this range; however, they should not be necessarily assumed as unsuitable regions because the significantly lower velocities in these regions can provide resting zones and refuge for many species. By increasing the grid size to 0.24 m and applying a 14.7% drop in *M*_*2*_ values, which was mentioned earlier as the average reduction due to increasing the grid size to the largest, no significant change in the spatial pattern of *M*_*2*_ values was observed and they mostly remained in 4–14 m^−1^ range as before. Small-scale regions with high *M*_*1*_ and *M*_*2*_ values which are adjacent to higher velocity zones may provide a suitable zone for place-specific activities, in which fish such as juvenile salmon and steelhead can forage while minimizing the bioenergetics cost of swimming^19,26^. Boulder placement, especially with higher densities, resulted in the more frequent appearance of such areas in this study.

For a complex region between boulders with several brown trout redds, it was reported that *M*_*4*_ values were greater by two orders of magnitude from a nearby region with a homogenous flow and without any redds^15^. In this study, after boulder placement, the *M*_*4*_ values increased by only one order of magnitude, and scenarios with the highest boulder concentration, S4-L, and S4-H, resulted in the largest *M*_*4*_ values, which indicated more flow complexity. It was indicated that *M*_*4*_ = 0.5 s^−1^ might be an upper threshold for brook trout (*Salvelinus fontinalis*) habitat^9^. In this study, after boulder placement, *M*_*4*_ values for all the arrangements exceeded 0.5 s^−1^. However, unlike the *M*_*4*_ values for the default grid size (0.06 m), for a larger grid spacing of 0.24 m, the *M*_*4*_ values mainly remained under 0.5 s^−1^.

It should be noted that habitat availability and selection may not be attributed to only the spatial flow patterns and subsequently the habitat hydraulic complexity metrics. There are several other factors such as cover, temperature, substrate composition, food availability, etc., that may substantially affect the instream habitat selection; however, considering the effects of all parameters together is difficult as they vary significantly in different sites^7^. For instance, substrate composition has a substantial influence on instream preferred habitat^35,60,61^. In this work, the bed was not movable which resulted in simplifying the in-situ conditions as different local hydraulics and substrate compositions are expected in a mobile bed around large roughness elements. In addition, other works have evaluated the habitat hydraulic complexity metrics over a variety of bed material sizes. These dissimilarities reduce the reliability of comparisons across studies and, again, highlight the need to consider a wider range of parameters with both hydraulic and structural complexities in relation to instream habitat for future work. More field data or experimental data with presence of live fish are needed to establish strong correlations between habitat hydraulic complexity metrics and influential factors for instream habitat assessment such as fish density, and availability of spawning grounds or feeding zones. Additionally, for a more accurate and useful comparison, based on the target species and their life stage as well as studied reach features, the effects of grid spacing should be considered and reported, if feasible.

## Conclusions

A series of experiments with varying flow rate and boulder concentration were performed to understand the effects of boulder placement (rock-ramp arrangement) on the kinetic energy gradient (*M*_*1*_ and *M*_*2*_) and modified recirculation (*M*_*4*_) metrics. It was found that based on the relevant ecological scale in a study (e.g., target fish length, or size of large roughness elements in the stream), an appropriate grid spacing should be carefully selected because it specifically affects average *M*_*1*_ and *M*_*4*_ in a reach with boulders. Boulder placement with even the lowest concentration (λ = 3.4%) significantly increased the average habitat hydraulic complexity metrics and varied the spatial distribution of the metrics in the detailed measurement zone. This also indicated that the metrics were able to represent flow complexity as expected. Assuming the studied metrics as the indicators of the hydraulic complexity of an instream habitat, the highest structural complexity, i.e., the highest boulder concentration (λ = 8.3%), generally resulted in the highest complexity. The effect of flow rate on the habitat complexity was not straightforward and was mostly statistically insignificant (except for *M*_*1*_). Testing a wider range of flow rates may clarify the effects of flow rate on the metrics investigated. The appearance of isolated, extremely large *M*_*2*_ values in the scenarios with boulders highly affected the average *M*_*2*_ in the studied area. It was therefore difficult to compare each scenario by utilizing the average *M*_*2*_ values as an indicator of habitat complexity.

The proposed relationships to predict the habitat hydraulic complexity metrics based on the average flow depth and velocity, flow rate, the ecologically relevant grid spacing, and boulder concentration in reaches with boulders (rock-ramp arrangement) may be helpful and practical for ecological restoration projects, which incorporate boulder placement. However, before the application of these relationships, their limitations and applicable range should be considered. They reflect the kinetic energy gradient metrics only in the longitudinal direction and are derived from a limited number of influential factors over a limited range. To improve the applicability of the proposed relationships, it is recommended that future studies use a wider range of the studied parameters and incorporate factors such as boulder configuration, boulder embedded depth, and substrate composition that have a potentially significant influence on the local flow field and habitat availability.

Based on the assessments from the available literature, boulder placement, especially at higher concentrations, may provide suitable habitats for different fish species in various life stages. Eventually, in ecological restoration projects with boulder placements, a boulder arrangement similar to the scenario with λ = 8.3% (S4-L and S4-H) may be a good candidate to restore habitat hydraulic complexity. This recommendation is based on the assumption that a higher habitat complexity in an instream habitat, generally leads to higher biotic diversity and is more beneficial for a variety of instream species including macroinvertebrates and fish. However, if the habitat complexity exceeds a certain threshold, it might not be necessarily suitable for some species; therefore, more studies linking instream species and their desirable habitat complexity are needed. Finding an optimum boulder concentration, at which the habitat complexity is maximized or optimized for a certain species, requires testing higher boulder concentrations and was not achieved in this study. However, it should be noted that considering significantly higher boulder concentrations than the ones examined in this study, might be impractical because they may disrupt fish passage or act as a bed rather than large roughness elements within the flow.

## Data Availability

The datasets generated during and/or analysed during the current study are available from the corresponding author on reasonable request.
